# Fusion of Heart Rate, Respiration and Motion Measurements from a Wearable Sensor System to Enhance Energy Expenditure Estimation

**DOI:** 10.3390/s18093092

**Published:** 2018-09-14

**Authors:** Ke Lu, Liyun Yang, Fernando Seoane, Farhad Abtahi, Mikael Forsman, Kaj Lindecrantz

**Affiliations:** 1 School of Engineering Sciences in Chemistry, Biotechnology and Health, KTH Royal Institute of Technology, Hälsovägen 11C, 141 57 Huddinge, Sweden; liyuny@kth.se (L.Y.); farhad.abtahi@ki.se (F.A.); mikael.forsman@ki.se (M.F.); 2 Institute of Environmental Medicine, Karolinska Institutet, Solnavägen 1, 171 77 Solna, Sweden; kaj.lindecrantz@ki.se; 3 Department of Clinical Science, Intervention and Technology, Karolinska Institutet, Hälsovägen 7, 141 57 Huddinge, Sweden; fernando.seoane@ki.se; 4 Swedish School of Textiles, University of Borås, Allégatan 1, 501 90 Borås, Sweden; 5 Department of Biomedical Engineering, Karolinska University Hospital, 1, 171 76 Solna, Sweden

**Keywords:** energy expenditure, wearable device, accelerometer, impedance pneumography, neural network

## Abstract

This paper presents a new method that integrates heart rate, respiration, and motion information obtained from a wearable sensor system to estimate energy expenditure. The system measures electrocardiography, impedance pneumography, and acceleration from upper and lower limbs. A multilayer perceptron neural network model was developed, evaluated, and compared to two existing methods, with data from 11 subjects (mean age, 27 years, range, 21–65 years) who performed a 3-h protocol including submaximal tests, simulated work tasks, and periods of rest. Oxygen uptake was measured with an indirect calorimeter as a reference, with a time resolution of 15 s. When compared to the reference, the new model showed a lower mean absolute error (MAE = 1.65 mL/kg/min, R^2^ = 0.92) than the two existing methods, i.e., the flex-HR method (MAE = 2.83 mL/kg/min, R^2^ = 0.75), which uses only heart rate, and arm-leg HR+M method (MAE = 2.12 mL/kg/min, R^2^ = 0.86), which uses heart rate and motion information. As indicated, this new model may, in combination with a wearable system, be useful in occupational and general health applications.

## 1. Introduction

The energy expenditure (EE), as an indicator of metabolic state and physical activity level, provides valuable information that can be used for occupational health and safety design [1], exercise, and daily life management, and prevention and treatment of health problems such as obesity and diabetes [2]. Direct measurement methods of EE or oxygen consumption (VO_2_), a commonly-used indicator of EE, requires expensive and sophisticated equipment, such as the direct calorimetry using metabolic chamber, the double labeled water method, and indirect calorimetry with a face mask, which are not suitable for daily free-living use [3]. Therefore, indirect measurement techniques using wearable sensors are desired, and have attracted significant attention in the last two decades; consequently, considerable effort has been allocated to the issue [4,5,6,7,8,9,10,11,12,13,14,15,16,17,18,19,20,21,22,23,24,25,26,27,28,29,30,31,32].

Heart rate (HR) monitoring is often used to estimate EE, as it has a good linearity with oxygen consumption in a large range of aerobic work [13,21]. The relationship between HR and EE at an individual level can be established through a calibration procedure, i.e., maximal or submaximal tests performed with a treadmill or cycle ergometer, which requires time and resources [33]. However, the poor relationship between HR and EE in resting and low intensity activities is an important limiting factor [24]. The HR-VO_2_ relation can vary in different activities [19], e.g., difference has been reported between upper body and lower body activities [34]. In addition, HR is affected by several factors that are not directly related to metabolism e.g., mental stress, emotions, and medication [16].

Accelerometry is also a popular tool to estimate physical activity related EE in free-living conditions. With count-based methods [11,35], the activity count is calculated using acceleration, and then directly linked to EE, while the type of activity being performed is not considered [6]. In activity related methods [4,7,12], firstly, the activity recognition is preformed, then the EE is estimated through a look-up table or by using the activity specified EE model [6]. The acceleration (ACC) measurement directly reflects the movement information. However, it lacks the information about the effort of the movements, which limits its effectiveness for assessing complex activities involving interaction with other objects, such as manual handling. Several methods that utilize HR and ACC have been proposed, which improves the estimation of EE by the sole use of HR or ACC [9,36].

Respiration is another factor that is related to EE [14]. Several studies have demonstrated that pulmonary ventilation (V_E_) has better linearity with EE compared to the HR [37,38]. As an accurate V_E_ measurement requires devices with facemasks or mouthpieces, the real application is very limited in free-living conditions. Recent developments in wearable technologies, such as impedance pneumography (IP), inductive plethysmography, and piezoresistive pneumography integrated in smart clothing [39,40,41,42,43], give new opportunities to use portable respiration measurement devices for EE estimation in a free-living setting, and preliminary studies have been carried out [15,18].

The purpose of this study was to develop and test a method that uses a combination of information from measurements of heart rate, respiration, and accelerations to estimate energy expenditure. The measurements were acquired through a wearable sensor system, and integrated by a model based on neural network. The wearable sensor system was developed under our research projects towards automatic risk assessment at work [44,45]. A lab experiment was implemented to support the development of the model and evaluate the developed system and estimation model. The proposed method was compared with two existing methods: HR-flex [28], a HR based method that uses a bi-linear model to improve the estimation in low intensity, and Arm-Leg HR+M [29,36], a method which uses combined HR and ACC measurements, with independent arm and leg calibration. The results showed improved accuracy over the two existing methods. In addition, the proposed method does not require complex lab calibration, which can dramatically improve the usability of such a system in field settings.

## 2. Materials and Methods

### 2.1. The Wearable Sensor System

**Figure 1:**
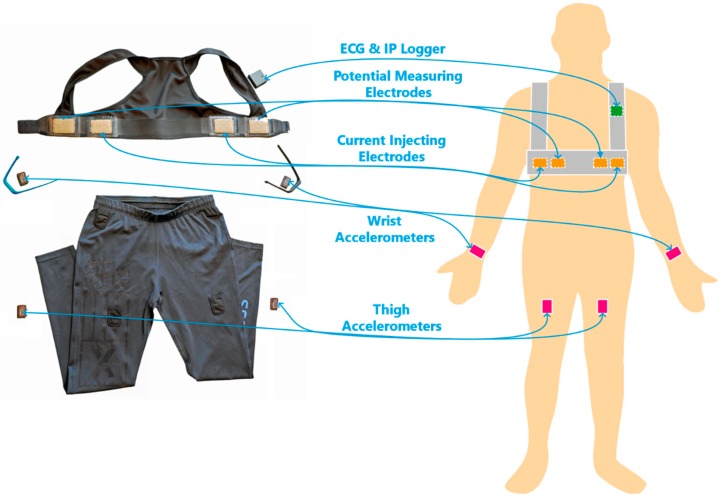
The wearable sensor system and its placement. The system includes a vest with textile electrodes, a wireless ECG and IP recording unit, 4 accelerometers, rubber wristbands, and trousers with specially designed pockets.

The wearable sensor system and the sensor placement are shown in Figure 1. The vest, reported in [40,46], includes four textile electrodes made by conductive fabric. One pair of electrodes was used for IP current injection, and the other was used for electric potential sensing for IP and ECG. A compact recorder, ECGZ2 (Z-Health Technologies AB, Borås, Sweden), for ECG and electrical bioimpedance was connected to the vest and placed in a pocket on the shoulder strap of the vest. The frequency of the injection current for impedance measurement was 50 kHz. ECG and IP signals were recorded with sampling rates of 250 Hz and 100 Hz, respectively. Four 3-axis accelerometers (AX3, Axivity Ltd., Newcastle, UK) were placed on both wrists, using rubber wristbands, and on the thighs, using trousers with specially designed pockets to hold the accelerometer units. The acceleration was recorded at 100 Hz.

### 2.2. Data Collection

#### 2.2.1. Participants

Nine men and three women participated in the laboratory experiment implemented in GIH, the Swedish School of Sport and Health Sciences, Stockholm, Sweden. The subjects consisted of a homogeneous group with young male subjects, and a heterogeneous group with both male and female participants in different age groups. Data from one subject was removed from the analysis because of the lack of a vest with a suitable size for the participant, which resulted in poor ECG and IP signal quality. The detailed characteristics of the included participants are shown in Table 1. All participants provided written informed consent. Ethical approval for the study was obtained from the Regional Ethical Review Board in Stockholm (Dnr 2016/724-31/5). 

#### 2.2.2. Experiment Protocol

The participants were asked to avoid intense physical activity for 1 day before the experiment, and to refrain from eating, smoking, drinking tea, coffee, or alcohol for at least 2 h beforehand. The experiment process took about 3 h. During the experiment, VO_2_ was measured by a computerized metabolic system (Jaeger Oxycon Pro, VIASYS Healthcare GmbH, Würzburg, Germany), where a facemask was worn by the participants. The experiment protocol consisted of three categories of activities: resting, simulated working tasks, and submaximal tests. The list of performed tasks and corresponding VO_2_ levels measured in the experiment is presented in Table 4 under the result section. After each task, the subject had a break for 5 to 25 min, until the HR returned to within 10 percent of the resting HR.

The resting test included resting in three postures: 20 min in lying, 5 min in sitting and 5 min in standing. During the resting test, the resting energy expenditure (REE) was measured. Five different working tasks, with different intensity levels and active muscle groups, were performed afterwards. Each of the tasks lasted 8–10 min. The office work required the participant to type on a computer while sitting beside a table. The painting work required the participant to simulate painting a wall at their own pace using a painting pole. The postal delivery work was performed by cycling at a cycle ergometer with 0.75 kg resistance. The meat cutting work was simulated by pulling a resistance band repetitively. The construction work included arm and whole body lifting tasks. The submaximal tests session consisted of 3 tests. The first was the Chester step test [47], with maximal 5 levels of incremental stepping pace. The second was a walking pace treadmill test as described in [36]. Each level of the treadmill test lasted three minutes. The speed was increased after the first level. From the second level, the inclination was raised by 2% between each stage. The third test was an arm ergometer test with a constant cadence while the resistance increased between each level [36]. All the submaximal tests were terminated when the HR of the subject reached the 80% of the age-predicted maximal HR (220 − age).

### 2.3. The Model for VO_2_ Estimation

**Figure 2:**
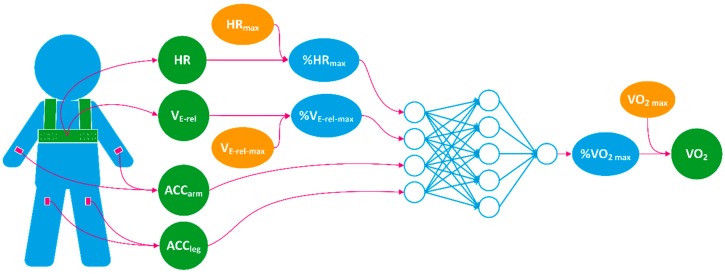
A demonstration of the flow of the oxygen consumption (VO_2_) process. The input and output are explained in Table 2.

The process of the estimation is shown in Figure 2. A multilayer perceptron neural network (MLPNN) with four input units, five hidden units, and one output unit was used to construct the model. The activation function of the hidden layer was hyperbolic tangent sigmoid function, and linear function for the output layer. All features and the output are listed in Table 2. All data were analyzed with 15-s non-overlapping windows. Four features were used that represent HR, V_E_, arm motion and leg motion, respectively. HR, V_E_, and VO_2_ were normalized by corresponding individual characteristics before being used as the inputs and output of the MLPNN to train a network with good genericization that learns characteristics at the group level.

The VO_2_ measurements were normalized by the individual maximal oxygen uptake (VO_2 max_), which was estimated through the Chester step test with pre-estimated VO_2_ level on each stage [47]. The HR was normalized by individual maximal HR (HR_max_), calculated by HR_max_ = 220 − age. The relative tidal volume (V_T-rel_) of each breath was represented by the impedance difference in peak and valley pairs of the filtered IP signal. The relative ventilation (V_E-rel_) during each 15-s epoch was acquired by the sum of the V_T-rel_ values in the window. A quadratic relationship between HR and V_E-rel_ was established for each subject by the least square method using measured HR and V_E-rel_ during the experiment. The maximal relative ventilation (V_E-rel max_) was then estimated by applying the HR_max_ to the HR-V_E-rel_ relationship. V_E-rel_ was then normalized by the V_E-rel max_ and fed to the network. The acceleration data was first band pass filtered with a 0.25−6 Hz passband; then, the mean absolute acceleration was computed for each 15-s epoch. For the arm and leg acceleration, the higher value from the right and the left sides of each epoch was picked.

### 2.4. Model Training and Cross Validation

The so-called Leave one subject out (LOSO) validation method was used. In repeated trials, all data except one subject was used for training the model; the data of that subject was used for testing the model. The LOSO method avoids test results that are overfitted to individual characteristics. The overall performance of the network was evaluated by combining test results from all LOSO cross validation. The training data was split for training and validation set with a ratio of 6:4, and the Levenberg-Marquardt backpropagation was used for the training process.

### 2.5. Comparision to Published Methods

Results from our method were compared with two published methods, i.e., HR-flex [28], one of the mostly used HR based method in the field, and Arm-Leg HR+M method [29,36], a method showed improved accuracy during occupational tasks in our previous evaluation [48]. The inputs and calibration requirements of all methods are listed in Table 3.

The flex-HR method [28] considers the nonlinearity in HR-EE relation in low intensity. It uses REE when the HR is below the flex point, and a linear HR-EE relationship when the HR is above the flex point. For the comparison, we chose to use step test data with pre-estimated VO_2_ levels on each stage for calibration, as it required the same level of test equipment as the new method. The REE was measured during the resting test. The flex-point was chosen as the average of the highest HR during rest and the lowest HR during walking on treadmill test.

The Arm-Leg HR+M method [29,36] accounts for the difference in HR-EE response between the upper and the lower body. It uses the level of arm and leg ACC and their ratio to determine the arm specified HR-EE equation, the leg specified HR-EE equation, or the REE for EE estimation. We used a treadmill test and arm ergometer test data to establish the arm and leg calibration respectively, together with a simultaneously measured VO_2_ level. The calibration requires a treadmill, an arm ergometer, and indirect calorimetry. Thresholds for the ACC level and ratio were re-adapted to our measurement data, as a different accelerometer and acceleration signal processing procedure were used in comparison to the original study.

### 2.6. Statistics

Estimated VO_2_ in 15-s epochs were compared to the criterion measurements. Bias, the mean absolute error (MAE), the root-mean-square-error (RMSE) and the coefficient of determination (R^2^) were calculated to evaluate the performance. Paired *t*-tests were performed to compare the absolute errors between the new method and each of the two published methods. Bland-Altman plots with error histograms were plotted to assess the agreement and the error distribution. 

## 3. Results

The mean levels of measured VO_2_ for performing each task during the experiment are listed in Table 4.

The training and testing results (%VO_2 max_) on each subject, as well as the averaged results from the LOSO validation, are shown in Table 5. The RMSE and R^2^ level from training and testing results were very close, which indicates the method has a good generalization among the participants. The averaged group bias was very low (−0.16%). However, a relatively lager bias (maximal 2.71%) can be found on individual level in few occasions. No strong relationship was found between the estimation errors and the personal characteristics, such as gender, age, and aerobic capacity.

The results of overall performance in VO_2_ estimation, measured by individual bias (IB), group bias (GB), MAE, RMSE, and R^2^ of three methods, are shown in Table 6. The proposed method showed a more accurate estimation (IB = 0.42 mL/kg/min, GB = −0.01 mL/kg/min, MAE = 1.65 mL/kg/min) compared to the flex-HR method (IB = 1.11 mL/kg/min, GB = 0.69 mL/kg/min, MAE = 2.83 mL/kg/min), where estimation error, individual bias, and group bias were significantly reduced (*p* < 0.001). The proposed method also showed a significant improvement (*p* < 0.001) in estimation error over the arm-leg HR+M method (MAE = 2.12 mL/kg/min).

**Figure 3:**
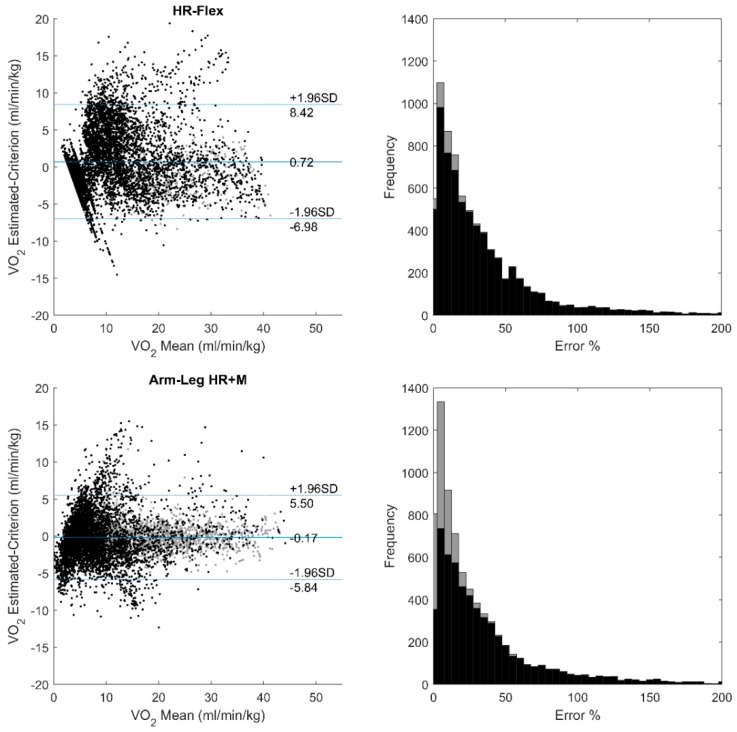

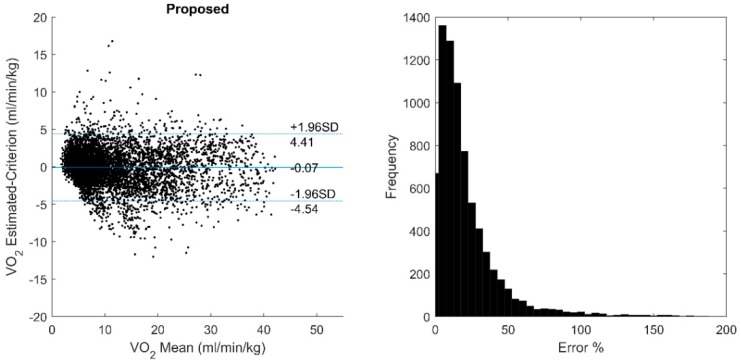
Bland-Altman plots and error rate histograms of flex-HR, arm-leg HR+M, and proposed methods against the criterion measurement. Data used in individual calibration are plotted in grey.

The Bland-Altman plots and the error rate histograms of three methods are shown in Figure 3. The proposed method shows a large improvement in the low intensity region. The mean estimation error rate was also reduced (28.1%) compared to the other methods (44.1% and 38.4% respectively).

The errors with each specific activity are shown in Table 7, where for each activity, the worst performance among the three methods is shown in bold and italic. The proposed method has a good overall generalization over different kinds of activities, except that a large bias on the simulated construction work can be found. Comparing to the flex-HR method, the error caused by different HR response to arm and leg activity was reduced in the proposed method by learning from group characteristics without arm calibration, which can be seen from the arm ergometer results, as well as from the top right corner of the Bland-Altman plot in Figure 3.

## 4. Discussion and Conclusions

In this study, we have demonstrated a method for free-living energy expenditure estimation that combines the HR, respiration, and motion information using nonlinear data driven modeling. In the experiment, the method showed improved accuracy over two established methods, based on HR and HR combined with ACC.

The method has also improved the usability by avoiding a complex laboratory calibration. The Chester step test used for VO_2 max_ estimation only requires a step with designed height, and takes only 6 to 10 min, which can be easily applied in the field. For certain ergonomic applications that use per cent maximum aerobic capacity (%VO_2 max_) as a measure of physical workload, the output of the network can be used directly without the need of multiplying individual VO_2 max_ value; hence, no calibration procedure is required. The wearable system used in the study is light-weight and easy to wear, which opens up the possibility for long-term, unobtrusive monitoring in different contexts. However, different contexts will come with different needs regarding number of accelerometers and their placement. The most versatile system would have many accelerometers at different sites on the body, but many sensors will increase the overall price of the system. Obviously, there will be trade-off between versatility, complexity, and cost. 

A method using neural network based model to estimate EE from HR has been reported previously in [31,32]. This method uses not only the HR, but also heart rate variability derived respiration rate, and HR ‘on and off dynamics’ as input features. However, very limited information has been shown about the implementation. Hence, we were not able to compare our method with it.

In previous studies [15,18], which used portable indirect respiration monitoring devices to estimate EE, the measured physical quantities such as transthoracic impedance and thoracic circumference distance were converted into flow or volume through a personal calibration process using a spirometer. In this study, a rough calibration of the personalized impedance level was acquired by using simultaneously-measured HR values. How much data is needed to establish a reliable relationship and the durability of the relationship should be further studied. In the experiment, we found our V_E-rel_ measurement through IP did not have very high linearity with the V_E_ measured by the indirect calorimetry. Possible causes for this discrepancy include the configuration of electrode position, posture change that alters the shape of ribcage [49], and motion artifacts. Applying optimized IP electrodes position [50] and advanced processing methods will have the potential to improve the IP measurement hence the EE estimation.

Limitations of this study include a small sample size (11 subjects), and the fact that limited activity types were performed under laboratory condition. The method has not yet been validated for complex real free-living scenarios, and the trained network could be overfitted to the activities that were performed in the experiment. The experiment has not taken into consideration many nonmetabolic-related factors that may alter HR or V_E_, such as mental stress and temperature. 

Since the new model showed a higher level of agreement with the reference methods compared to two existing methods, this study indicates a high potential for applying information fusion of HR, respiration, and motion data in combination with a nonlinear statistical learning method in the field of unobtrusive energy expenditure estimation. The solution may be used both in occupational and general health applications. Studies with improved respiration monitoring techniques and varied populations with larger size under free-living conditions are suggested in future development.

## Figures and Tables

**Table 1:** Characteristics of included participants (median [range]).

	Men (*N* = 9)	Women (*N* = 2)	All (*N* = 11)
Age (year)	27 [21–65]	43 [25–61]	27 [21–65]
Height (cm)	181 [171–199]	169 [165–173]	177 [165–199]
Weight (kg)	77 [51–89]	60 [58–62]	75 [51–89]
BMI (kg/m^2^)	22.8 [17.4–25.6]	20.9 [20.7–21.2]	22.6 [17.4–25.6]
VO_2 max_ (mL/min/kg)	42.9 [32.1–54.6]	35.6 [30.9–40.3]	40.3 [30.9–54.6]

**Table 2:** Summary of the input features and the output of the neural network.

Input Features	% HR_max_	HR normalized by age predicted HR_max_
	% V_E-rel max_	V_E-rel_ normalized by estimated V_E-rel max_
	ACC_arm_	Mean absolute value of wrist acceleration
	ACC_leg_	Mean absolute value of thigh acceleration
Output	% VO_2 max_	VO_2_ normalized by estimated VO_2 max_

**Table 3:** A comparison of requirements of input data and personalized measurement among the three methods.

Methods	Input Data	Additional Individualized Measurements
Flex-HR	HR	Flex HR Point REE HR-VO_2_ Calibration
Arm-Leg HR+M	HR, ACC_leg_, ACC_arm_	REE Leg HR-VO_2_ Calibration Arm HR-VO_2_ Calibration
Proposed	HR, ACC_leg_, ACC_arm_, V_E-rel_	VO_2 max_

**Table 4:** A summary of tasks performed during the experiments and corresponding mean VO_2_ level (mL/min/kg) of the 11 subjects.

Group	Task	VO_2_ Level (Mean ± SD)
Resting	Lying	3.78 ± 0.96
	Sitting	3.82 ± 1.16
	Standing	4.01 ± 0.41
Work Tasks	Office Work	4.01 ± 1.42
	Painting Work	8.51 ± 1.68
	Postal Delivery Work	14.04 ± 2.37
	Meat Cutting Work	7.62 ± 1.89
	Construction Work	12.24 ± 4.56
Submaximal Tests	Step Test	22.23 ± 7.71
	Treadmill Test	22.88 ± 8.05
	Arm Ergometer Test	11.06 ± 4.98

**Table 5:** Results of the cross validation of the relative VO_2_ (%VO_2 max_) from the neural network.

Gender	Age (Year)	Weight (kg)	Height (cm)	BMI (kg/m^2^)	VO_2 max_ (mL/kg/min)	%VO_2 max_					
						Train			Test ^1^		
						Bias	RMSE	R^2^	Bias	RMSE	R^2^
M	65	80	188	22.6	32.7	−0.03	5.26	0.92	−2.03	5.74	0.88
M	21	77	176.5	24.7	54.6	0.07	5.54	0.90	−0.35	5.40	0.92
F	61	62	173	20.7	30.9	0.10	5.06	0.92	−1.19	8.03	0.84
F	25	58	165.5	21.2	40.3	−0.01	5.39	0.91	0.11	4.55	0.93
M	27	88.5	199	22.3	47.8	0.04	5.33	0.91	0.18	4.69	0.94
M	27	51	171	17.4	39.6	0.14	5.21	0.92	−0.36	6.61	0.87
M	25	79.8	176.5	25.6	43.6	0.05	5.35	0.91	1.84	4.60	0.93
M	29	88.9	190	24.6	42.9	−0.03	5.54	0.91	−0.51	4.07	0.95
M	42	75	177	23.9	32.1	0.03	5.26	0.92	2.71	5.93	0.88
M	26	75	181.5	22.8	37.2	0.06	5.28	0.91	−0.68	4.86	0.92
M	26	68.5	184	20.2	44.8	0.08	5.30	0.91	−1.47	5.76	0.90
Average Mean (SD)									−0.16 (1.38)	5.47 (1.13)	0.91 (0.03)

^1^ In each row, the data for the specific subject was excluded in the training and used for the testing.

**Table 6:** Comparison of VO_2_ estimation results among flex-HR, arm-leg HR+M, and proposed method (mL/kg/min).

Methods	Individual Bias ^1^	Group Bias	MAE	RMSE	R^2^
Flex-HR	1.11	0.69	2.83	4.00	0.75
Arm-Leg HR+M	0.60	−0.09	2.12	2.95	0.86
Proposed	0.42	−0.07	1.65	2.28	0.92

^1^ Mean absolute value of individual biases.

**Table 7:** Comparison of task specific errors among three methods (mL/kg/min).

	Resting	Office Work	Painting	Postal Delivery	Meat Cutting	Construction Work	Step	Treadmill	Arm Ergometer
**Flex-HR**									
Bias	−0.05	−0.29	−0.47	***−2.42***	***1.84***	1.05	−1.05 ^1^	***−0.81***	***4.18***
RMSE	0.90	0.84	***3.89***	***4.15***	***4.33***	3.90	***2.85*** ^1^	***2.92***	***5.91***
**Arm-Leg HR+M**									
Bias	***−1.42***	***−0.93***	−***1.90***	−1.59	−0.16	−1.09	***−0.38***	−0.01 ^2^	0.00 ^2^
RMSE	***2.50***	***2.09***	2.55	2.57	1.56	***4.44***	2.53	1.82 ^2^	1.14 ^2^
**Proposed**									
Bias	0.02	0.17	−0.47	−0.46	0.55	***−2.01***	−1.08 ^3^	0.44	0.06
RMSE	0.93	0.86	1.69	2.36	1.62	3.88	2.83 ^3^	2.71	1.69

The bold and italic numbers indicate the largest error in each activity. ^1^ Data used for individual calibration, with pre-estimated VO_2_ level. ^2^ Data used for individual calibration, with measured VO_2_ level. ^3^ Data used for VO_2max_ estimation, with pre-estimated VO_2_ level.
